# 676. Impact of Stratified Testing Algorithm Utilizing Rapid Testing and Polymerase Chain Reaction (PCR) Tests for Viral Infections

**DOI:** 10.1093/ofid/ofab466.873

**Published:** 2021-12-04

**Authors:** Akshay M Khatri, Rehana Rasul, Molly McCann-Pineo, Rebecca Schwartz, Aradhana Khameraj, Prashant Malhotra, Bruce Farber

**Affiliations:** 1 Donald and Barbara Zucker School of Medicine at Hofstra/Northwell Health, Glen Oaks, New York; 2 Feinstein Institute of Medical Research Northwell Health, Manhasset, New York; 3 Department of Occupational Medicine, Epidemiology and Prevention, Feinstein Institute for Medical Research, Donald and Barbara Zucker School of Medicine at Hofstra-Northwell, Manhasset, New York; 4 Northwell Health, Manhasset, New York; 5 Northshore University Hospital Northwell Health, Manhasset, New York; 6 Northshore University Hospital Northwell Health, Manhasset, NY United States, Manhasset, New York

## Abstract

**Background:**

In 2017, the multiplex respiratory viral panel (RVP) test was the only test available for patients (pts) with respiratory symptoms in our emergency department (ED). In 2018, the more rapid influenza/respiratory syncytial virus (Flu/RSV) test was incorporated in a stratified testing algorithm (STA) – depending on clinical features and physician discretion, pts underwent either Flu/RSV or RVP. We analyzed the STA impact by comparing data between winters of 2017 and 2018.

**Methods:**

In a retrospective, single-center cohort study in suburban NY, admitted pts ≥18 years diagnosed with viral infections (by either test) were included. We excluded pts diagnosed at another hospital, admitted to intensive care or observation (< 24 hours) units and pts with missing data. Data was collected through electronic medical chart review.

Primary outcomes were clinical evaluation time [time between triage and test order]; laboratory-turnaround (lta) time (between order and result); ED length of stay [EDLOS] (between admit order and bed assignment). Secondary outcomes included isolation time (between result to start of isolation precautions), treatment time (between result to influenza treatment). Outcome differences were assessed using Chi-Square and Mann-Whitney rank sum tests for categorical and continuous variables, respectively.

**Results:**

734 pts were included in the study [368 in 2017; 366 in 2018]. Median age was 75 years and 55.9% were female. After implementing the STA, EDLOS was significantly lower **[Table 1]**, with no significant differences in other parameters. Lta times were slightly higher after implementation [25 minutes (2017) v/s 29 minutes (2018)].

Table 1. Differences in clinical and laboratory turnaround times among patients admitted with viral infections in winters of 2017 and 2018

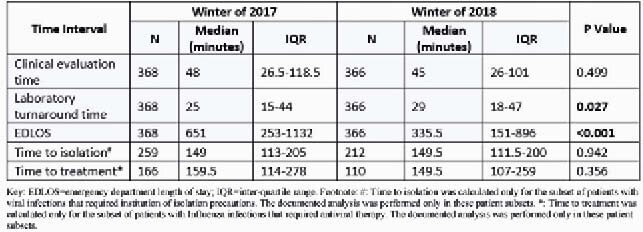

**Conclusion:**

A stratified diagnostic algorithm may have reduced EDLOS, but without significant differences in other outcomes. A higher lta time might have been due to testing constraints, heterogeneous pt populations or other confounders. Prospective studies will help assess the real-world impact of such algorithms.

**Disclosures:**

**Prashant Malhotra, MBBS, MD,FACP, FIDSA**, **Gilead Sciences** (Scientific Research Study Investigator, Other Financial or Material Support, Site PI for a industry funded multi center research study)

